# Identification of the Immediate-Early Genes of Cyprinid Herpesvirus 2

**DOI:** 10.3390/v12090994

**Published:** 2020-09-07

**Authors:** Ruizhe Tang, Liqun Lu, Beiyang Wang, Jiao Yu, Hao Wang

**Affiliations:** 1 National Pathogen Collection Center for Aquatic Animals, Shanghai Ocean University, Shanghai 201306, China; m180100211@st.shou.edu.cn (R.T.); lqlv@shou.edu.cn (L.L.); 2 Key Laboratory of Freshwater Aquatic Genetic Resources, Ministry of Agriculture Shanghai Ocean University, Shanghai 201306, China; 3 National Demonstration Center for Experimental Fisheries Science Education, Shanghai Ocean University, Shanghai 201306, China; 4 China Society of Fisheries, Beijing 100000, China; baye916@163.com (B.W.); yjsnlmc@163.com (J.Y.)

**Keywords:** cyprinid herpesvirus 2, immediate-early gene, *Carassius auratus gibelio*, *ORF121*

## Abstract

Cyprinid herpesvirus 2 (CyHV-2), which infects goldfish and crucian carp causing high mortality, is an emerging viral pathogen worldwide. The genome of CyHV-2 is large and comprises double-stranded DNA, including several genes similar to cyprinid herpesvirus 1, ictalurid herpesvirus-1, cyprinid herpesvirus 3, and ranid herpesvirus-1. Genes of DNA viruses are expressed in three temporal phases: immediate-early (IE), early (E), and late (L) genes. Viral IE genes initiate transcription as soon as the virus enters the host, without viral DNA replication. IE gene products enable the efficient expression of E and L genes or regulate the host to initiate virus replication. In the present study, five IE genes of CyHV-2 were identified, including open reading frame (ORF)54, ORF121, ORF141, ORF147, and ORF155. Time course analysis and reverse transcription polymerase chain reaction confirmed five IE genes, thirty-four E genes, and thirty-nine L genes. In addition, all 150 ORFs identified in the CyHV-2 genome are transcribed, and are expressed in chronological order, similar to other herpesviruses. This study is the first to identify the IE genes of CyHV-2, which will provide more information for viral molecular characterization.

## 1. Introduction

Cyprinid herpesvirus 2 (CyHV-2), a pandemic pathogen for *Carassius auratus,* (e.g., crucian carp and goldfish) is a large, enveloped DNA virus that causes herpes viral hematopoietic necrosis (HVHN) disease. HVHN disease causes acute gill hemorrhage and high mortality, leading to a serious threat to crucian carp and goldfish populations. The disease has spread across the world and can be found in Japan [1], the USA [2], the UK [3], China [4], New Zealand [3], and Australia [5]. CyHV-2 is a member of the *Alloherpesviridae* family (genus *Cyprinivirus*), which also comprises cyprinid herpesvirus 3 (CyHV-3) and carp pox virus (CyHV-1). Characteristically, CyHV-2 and CyHV-3 can establish acute, persistent, and latent infection in their host, similar to other herpesviruses [6]. Symptomless surviving fish in populations with persistent infection can promote viral spread, representing a major route for CyHV-2 transmission that affects the global trade of healthy and infected goldfish. Recently, the complete genomes of seven CyHV-2 isolates have been published, including ST-J1 (NC019495, 290,304 bp, 150 ORFs), SY-C1 (KM200722, 289,365 bp, 140 ORFs), SY (KT387800, 290,455 bp, 150 ORFs), YC-01 (MN593216, 275,367 bp, 150 ORFs), YZ-01 (MK260012, 288,076 bp, 148 ORFs), CNDF-TB2015 (MN201961, 288,321 bp, 181 ORFs), and CaHV (KU199244, 275,348 bp, 150 ORFs). The above complete genomes have improved our understanding of the biology of CyHV-2. However, the molecular pathogenesis of CyHV-2 remains poorly understood.

Like many other DNA viruses, herpesvirus genes are classified into three distinct phases: immediate-early (IE) genes, early (E) genes, and late (L) genes. Immediate-early genes play a key role in the regulation of E and L genes. In addition, many IE genes code for regulatory proteins that are essential to regulate the physiological state of the host cell to enhance viral replication [7]. Fifteen CyHV-3 IE genes were identified using a fibroblast-like cell line as an in vitro infection system together with cycloheximide (CHX) as an inhibitor [8]. However, among all sequenced alloherpesviruses, twelve genes (*ORF33, ORF46, ORF47, ORF61, ORF71, ORF72, ORF78, ORF79, ORF80, ORF90, ORF92* and *ORF107* referred as core genes) are conserved significantly [9]. The expression order of the open reading frames (ORFs) identified in CyHV-2 remains unknown.

In the present study, we identified 5 IE genes, 34 E genes, and 39 L genes from the CyHV-2 genome using high-throughput sequencing combined with CHX and Cytarabine (Ara-C) inhibitors, similarly to other herpesviruses. The transcription of IE genes, such as *ORF54, ORF121, ORF141* and *ORF155* of CyHV-2 started soon after infection. By 8 h, CyHV-2 had completed viral genome replication.

## 2. Materials and Methods

### 2.1. Cell Cultures and Virus Preparation

The RyuF-2 cell line was generously provided by Dr. Motohiko Sano (Tokyo University of Marine Science and Technology). This cell line, which was derived from a Ryukin goldfish caudal fin, can be infected with CyHV-2 and can be used for in vitro virus replication. These cells were maintained at 22 ℃ and cultivated in modified tissue culture Medium199 (Gibco, Grand Island, NY, USA). Fetal bovine serum (FBS; 10%; Gibco) and 2% Penicillin–Streptomycin (Gibco) were supplemented to the medium for stable cell culture. CyHV-2 was first isolated in Japan in 1999 from diseased goldfish [10]. Viruses were collected from infection RyuF-2 cells at seven days post-infection. Cells and cell debris were removed by centrifugation at 12,000× *g* for 20 min at 4 °C. The supernatant was filtered through a 0.22-µm filter unit to collect the virus. The virus titer was quantified by gradient dilution counting. The harvested virus was aliquoted and placed at −80 °C for subsequent experiments.

### 2.2. Cell Viability Test

The viability of RyuF-2 cells was tested using a Muse^®^ Count & Viability kit (Merck KGaA, Darmstadt, Germany) after treatment with different concentrations of inhibitors for 14 h (time elapsed after 4 h of pretreatment of cells, 2 h for CyHV-2 incubation, and 8 h post infection). The concentrations of CHX and Ara-C used in this test were 3 μg/mL, 6 μg/mL, 9 μg/mL, 12 μg/mL, 15 μg/mL, 30 μg/mL, 60 μg/mL, 90 μg/mL, 120 μg/mL, 150 μg/mL, and 30 μg/mL, 60 μg/mL, 90 μg/mL, 120 μg/mL, 150 μg/mL, 180 μg/mL, 210 μg/mL, 240 μg/mL, 270 μg/mL, 300 μg/mL. All cells and debris from three biological replicates in the wells were re-suspended in sterile filtered phosphate-buffered saline (PBS) at 10^6^ cells/mL. Following the manufacturer’s instructions, 50 μL of cell suspension was mixed with 450 μL of Count & Viability Reagent and placed in the dark for 5 min at room temperature. Using the Muse^®^ Cell Analyzer, we obtained cell viability and cell count data. To determine the correct parameters for Muse^®^ gating, a suspension of well-grown cells at 10^6^ cells/mL was used to set the viability profile and to determine the gates for the Muse^®^ Cell Analyzer and the Cell Count and Viability assay Kit.

### 2.3. Protein and DNA Synthesis Inhibitor Treatment of Cultured Cells

RyuF-2 cells were grown in six-well plates at 10^6^ cells/well. CHX was used to inhibit protein synthesis and cytosine-β-D-arabinofuranoside (Ara-C) was used to inhibit DNA synthesis (both Sigma, St. Louis, MO, USA). After 4 h of treatment with inhibitors, cells comprising the infection group were treated with CyHV-2 (MOI (Multiplicity of infection) = 1) for 2 h in the presence of the inhibitors. The virus was removed and the cells were overlaid with fresh media with the same concentration of added inhibitor. In all experiments, the cells at this stage were designated as being at time 0.

### 2.4. Nucleic Acid Extraction and Quantitative Real-Time Reverse Transcription PCR (qRT-PCR) Analysis

Total RNA from RyuF-2 samples (1 × 10^6^ cells) was extracted using 1 mL of the TRIzol Reagent (Invitrogen, Waltham, MA, USA), and subjected to purification using phenol/chloroform and ethanol precipitation. A Nanodrop instrument (ND-1000; Nanodrop Technologies, Wilmington, DE, USA) was used to quantify the RNA. A 5-μg sample of RNA was used to generate cDNA using PrimeScript RT Master Mix (Takara, Shiga, Japan) for quantitative real-time PCR (qPCR) and transcriptome sequencing. PrimeSTAR Max DNA Polymerase (Takara, Japan) was used to amplify 1 μL of the resultant cDNAs. Specific sets of primers for each ORF were designed using Primer Premier 5.1 (Premier Biosoft, Palo Alto, CA, USA; Table 1) according to the CyHV-2 genome sequence (GenBank: NC_019495) [11]. The reactions contained 25 µL of Prime STAR Max Premix (2×), 1 µM forward/reverse primers, and 500 ng of cDNA. Thermal cycling included one cycle of initial activation at 98 **°**C for 3 min, followed by 34 cycles at 98 **°**C for 10 s, 56 **°**C for 5 s, and 72 **°**C for 15 s, and a final extension at 72 **°**C for 10 min. PCR products (10 µL) were subjected to agarose gel electrophoresis to show that the molecular mass of each PCR product was related to the appropriate viral DNA sequence. Electrophoresis comprised a 1.5% agarose gel, 1 × TAE (40 mM Tris–acetate pH 8 and 1 mM EDTA), 2000 DNA Marker (Takara) and nucleic acid dye (Tanon™, China). *Actin* and ORFs of CyHV-2 were used as controls to check that the amplified genes were present among the viral transcripts. The primers derived from actin was used successfully for PCR with cDNA prepared from infected and non-infected cells as the template.

### 2.5. Electron Microscopy

The samples were collected for electron transmission as previously described [12]. According to Section 2.3, RyuF-2 cells were pretreated with the inhibitors at indicated concentration for 4 h, then incubated with CyHV-2 at an MOI of 1 for 2 h. The control group (without inhibitor), the CHX (12 μg/mL) treatment group, and the Ara-C (300 μg/mL) treatment group samples were observed 8 h post-infection. Slides were fixed in 4% paraformaldehyde for 30 min at room temperature and in cold methanol for 10 min at −20 °C. Transmission electron microscopy (TEM) images were obtained using a 7000-FA Transmission Electron Microscope (Hitachi, Tokyo, Japan).

### 2.6. Sequencing of the CyHV-2 Transcriptome and Bioinformatic Analyses

To acquire as many transcripts as possible and to minimize the loss of transcripts with low expression, the RNA was sequenced as three parallel samples. Total RNAs of the virus infection group, CHX treatment group and Ara-C treatment group cells were used for high-throughput sequencing by using the method described in Section 2.4. Transcriptome sequencing was performed using an Illumina HiSeq 4000 sequencer (Illumina San Diego, CA, USA) at Majorbio (Shanghai, China) according to the manufacturer’s instruction. The raw data was analyzed using fastx_toolkit_0.0.14 (http://hannonlab.cshl.edu/fastx_toolkit/) for quality assessment statistics to ensure that the quality scores were greater than 20 and the sequencing error rates were less than 0.1%. Then, we used SeqPrep (https://github.com/jstjohn/SeqPrep) and Sickle (https://github.com/najoshi/sickle) software to read the data, remove the linker sequence from the reads, and to discard sequences less than 30 bp after mass trimming, and reads with ambiguous bases ‘N’. The clean data were assembled from scratch using Trinity (https://github.com/trinityrnaseq/trinityrnaseq) to generate contigs and singletons [13]. The clean reads generated from the three libraries (Infection, CHX, Ara-C) were all mapped to the reference sequences, which comprised the unigenes assembled from the transcriptome data, using Bowtie2 [14]. CyHV-2 Sequence information was obtained from the ST-JI (GenBank accession no. NC_019495). Phylogenetic trees were built using the neighbor-joining method in MEGA 7 software [15], with the bootstrap value set to 1000. Alignment was performed using DNAMAN software (Lynnon Biosoft, San Ramon, CA, USA). The alloherpesviruses family sequence information was obtained from NCBI (National Center for Biotechnology Information) (SY: KT387800, ST-J1: NC_019495, SY-C1: KM200722, CaHV: KU199244, YC-01: MN593216, CyHV1: NC_019491, CyHV3: NC_009127, IcHV1 (Ictalurid Herpesvirus 1): NC_001493, RaHV2 (Ranid herpesvirus-2): NC_008210, RaHV1 (Ranid herpesvirus-1): NC_008211, and AngHV1 (Anguillid herpesvirus-1): FJ940765).

### 2.7. Analysis of CyHV-2 Replication Using PCR and qPCR

As previously described, after treatment with different concentrations of inhibitors, viral gene expression was measured using Prime STAR Max DNA Polymerase amplified cDNA. qPCR was performed to assess the temporal expression of selected CyHV-2 genes. The qPCR reactions contained 12.5 µL of TB Green Premix Ex Taq II (2×) (Takara, Japan), 0.4 µM forward/reverse primers, and 200 ng cDNA. The thermal cycling program comprised: 95 ℃ for 30 s and then 39 cycles of 95 ℃ for 5 s and 59 ℃ for 30 min. The qPCR reactions were repeated three times. To construct a standard curve, serial decimal dilutions were performed using DNA extracted from the virus, and all results were normalized to the relevant actin expression levels. The normalization calculation was performed using the standard procedure for absolute quantification. Statistical significance was calculated using one-way analysis of variance (ANOVA). Values were considered as significant (*) if they had a *p* value of 0.01 to 0.05, very significant (**) if they had a *p* value of 0.001 to 0.01, and extremely significant (***) if they had of *p* value 0.0001 to 0.001.

## 3. Results

### 3.1. Screening of CyHV-2 IE Genes

**Figure 1:**
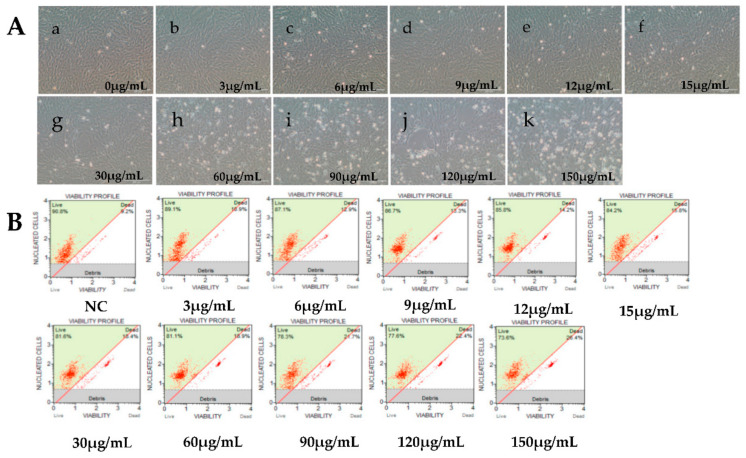
Effects of cycloheximide (CHX) at different concentrations on RyuF-2 cell viability. Cells were treated with CHX to inhibit protein synthesis in order to screen for immediately genes. Cell viability was determined under a microscope after 14 h of treatment with the different concentrations of CHX (5~150 μg/mL). (**A**, a–k), images of cell cultures under various CHX concentrations. (**B**) Corresponding cell viability graphs.

**Figure 2:**
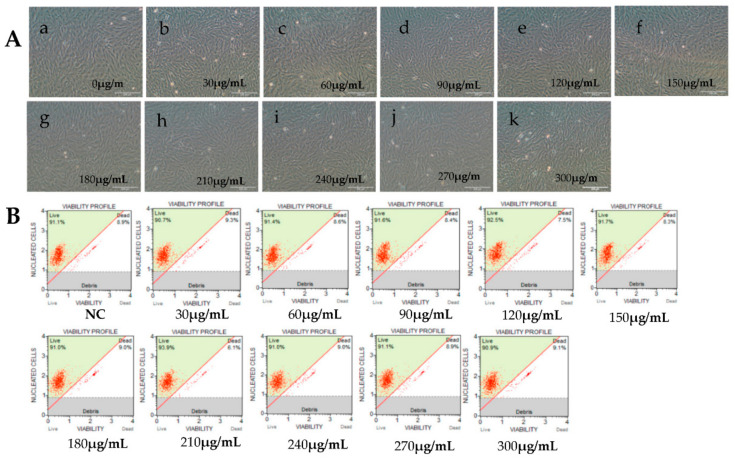
Effects of cytarabine injection (Ara-C) at different concentrations on RyuF-2 cell viability. Ara-C hinders the action of DNA polymerase and inhibits DNA synthesis. Cell viability was determined under a microscope after 14 h of treatment with the different concentrations of Ara-C (0~300 μg/mL). (**A**, a–k), images of cell cultures under various Ara-C concentrations. (**B**) Corresponding cell viability graphs.

**Figure 3:**
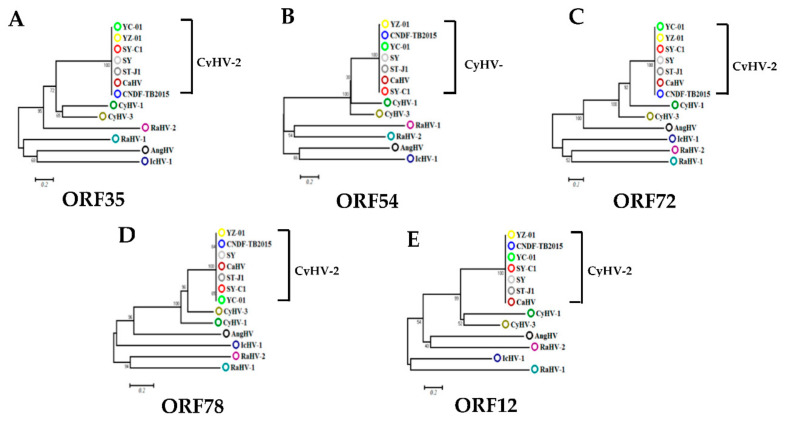
Phylogenetic analysis of five ORFs from CyHV-2. The Phylogenetic trees (**A**–**E**) were constructed using the neighbor-joining method in MEGA7 based on five ORFs from CyHV-2 (SY (GenBank accession no. KT387800), ST-J1 (NC_019495), SY-C1 (KM200722), CaHV (KU199244), YC-01 (MN593216)), CyHV1 (NC_019491), CyHV3 (NC_009127), AngHV1 (Anguillid herpesvirus-1; FJ940765), RaHV1 (Ranid herpesvirus-1; NC_008211), RaHV2 (Ranid herpesvirus-2; NC_008210), and IcHV1 (Ictalurid Herpesvirus 1; NC_001493). Branch lengths are proportional to evolutionary distance showing the divergence among different species.

**Figure 4:**
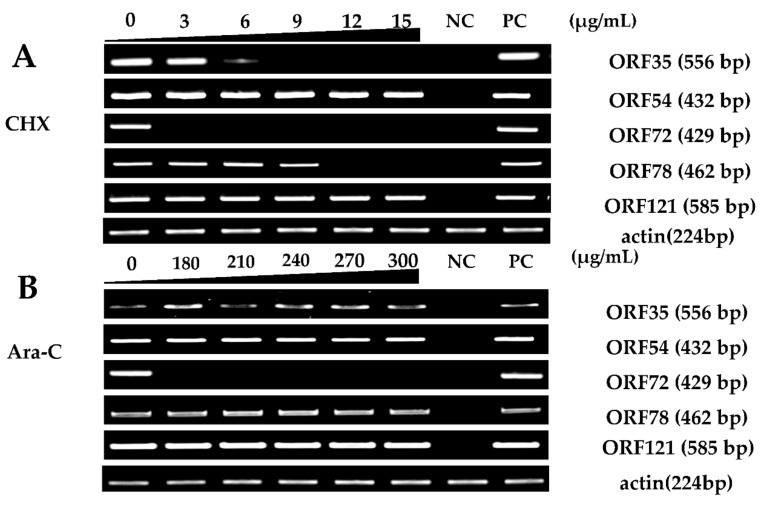
Effective concentration screening of the inhibitors. Gene expression differences of five selected CyHV-2 genes in infected RyuF-2 cells were obtained using Real-Time Reverse Transcription (RT-PCR) under different concentrations of CHX (0 to 15 μg/mL) (**A**) or Ara-C (0 to 300 μg/mL) (**B**) treatment. Treatment of infected cells with 12~15 μg/mL CHX or 180~300 μg/mL Ara-C inhibited the expression of certain genes. The gene encoding actin was used as an internal control. NC: negative control; PC: CyHV-2 DNA used as positive control.

Viral IE gene expression occurs soon after the virus enters the cell and does not need the expression of additional viral proteins. CHX was used to block new cellular protein synthesis, which would block the transcription of viral E and L genes, but not IE genes [12]. Ara-C acts by inhibition of DNA synthesis, thus blocking the transcription of viral L genes. Therefore, CHX combined with Ara-C could be used to distinguish viral IE, E, and L genes. To construct a screening model to identify viral IE genes, we first optimized the concentrations of CHX and Ara-C. The cytotoxic effects of CHX and Ara-C on RyuF-2 cells were investigated. Cell proliferation and viability were evaluated using a Muse Viability Assay Kit according to the manufacturer’s protocol. Figure 1 shows little cytotoxicity when the concentration of CHX was lower than 15 μg/mL. Moreover, none of the concentrations of Ara-C had obvious cytotoxic effect on RyuF-2 cells up to the highest concentrations tested (300 µg/mL) (Figure 2). Next, RyuF-2 cells were pre-treated for 4 h with Ara-C or CHX at different concentrations and then challenged with CyHV-2 in the presence of the inhibitors. RNA was extracted to assess viral gene transcription. Figure 3 shows by phylogenetic tree analysis that all five selected genes clustered in the same clade as CyHV-2. As shown in Figure 4A, *ORF35, ORF72*, and *ORF78* expression was inhibited in the samples treated with CHX at 12–15 μg/mL, whereas CHX-treatment did not affect *ORF54* and *ORF121* transcription. In addition, the transcription of *ORF72* was also inhibited by Ara-C at all test concentrations from 180 to 300 μg/mL, whereas Ara-C had no effect on *ORF35, ORF54, ORF78*, and *ORF121* expression (Figure 4B). The results from samples treated with CHX combined with Ara-C confirmed that *ORF72*, encoding a major capsid protein of CyHV-2, is a viral L gene. As shown in Figure 4, *ORF78* and *ORF121* were confirmed as CyHV-2 E and IE genes, which contrasted with CyHV-3. The above data showed that CHX (12 μg/mL) combined with Ara-C (300 μg/mL) could be used efficiently in RyuF-2 cells to screen for viral IE genes.

**Figure 5:**
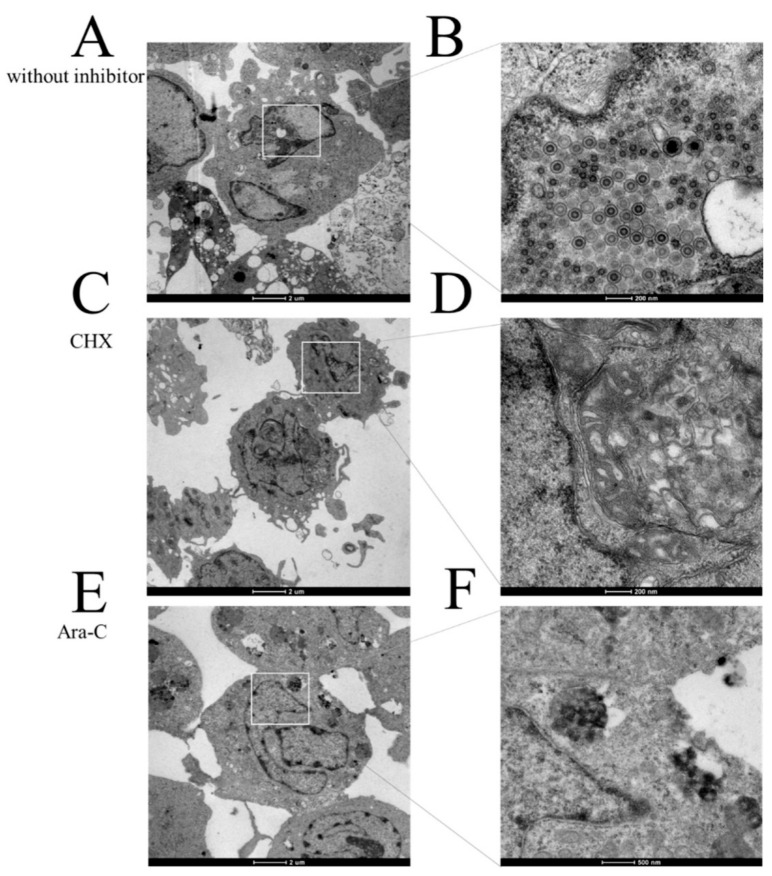
Action of CHX and Ara-C on progeny virus production. (**A**,**B**) Transmission electron microscopy image of the uptake of CyHV-2 into RyuF-2 cells (including a magnified image of a region of the first panel). (**C**,**D**) Transmission electron microscopy image of the uptake of CyHV-2 into RyuF-2 cells in the presence of CHX (including a magnified image of a region of the first panel). (**E**,**F**) Transmission electron microscopy image of the uptake of CyHV-2 into RyuF-2 cells in the presence of Ara-C (including a magnified image of a region of the first panel).

To confirm the effects of CHX and Ara-C on viral replication, infected cells treated with CHX (12 μg/mL) and Ara-C (300 μg/mL) before and at eight hours after infection were collected to investigate virus replication using transmission electron microscopy. As shown in Figure 5A,B, a large number of typical viral particles were observed in the RyuF-2 cells of the control group (without inhibitor). As expected, Figure 5C–F show no obvious particles in the inhibitor treatment groups compared with those in the control group. These data are consistent with qRT-PCR detection shown in Figure 4.

**Figure 6:**
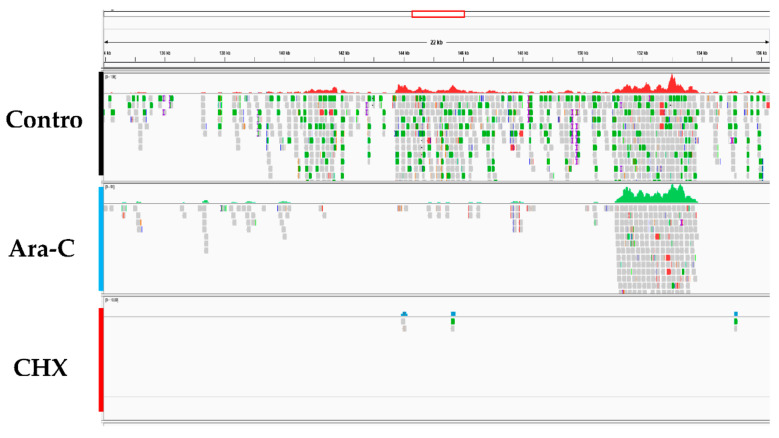
High-throughput sequencing data analysis. Panels show the differences in transcript expression levels among the groups after treatment with different inhibitors. Control: Infected CyHV-2 group; Ara-C: Infected with CyHV-2 and treated with cytarabine; CHX: Infected with CyHV-2 and treated with cycloheximi.

CyHV-2 ORF expression was examined for three groups: the control group (without inhibitor), the CHX (12 μg/mL) treatment group, and the Ara-C (300 μg/mL) treatment group. RyuF-2 cells at eight hours post infection with CyHV-2 were subjected to mRNA extraction followed by RNA-seq analysis, which produced several million high quality reads for each sample (Table 2). The cleaned reads were analyzed and mapped to the CyHV-2 genome. As shown in Figure 6, Table 2, and Supplementary Materials Table S1, all 150 annotated ORFs from CyHV-2 ST-J1 were completely sequenced in the control group, which indicated that CyHV-2 DNA synthesis had been achieved at eight hours post-infection. As expected, fewer ORFs were expressed in the CHX groups compared with those expressed in the Ara-C group (61 vs. 127) (Figure 6 and Table S1). Thus, these 61 ORFs were identified as candidate IE genes in CyHV-2. Table S1 provides detailed information concerning the candidate IE genes.

### 3.2. CyHV-2 IE Genes Confirmation Using qRT-PCR

**Figure 7:**
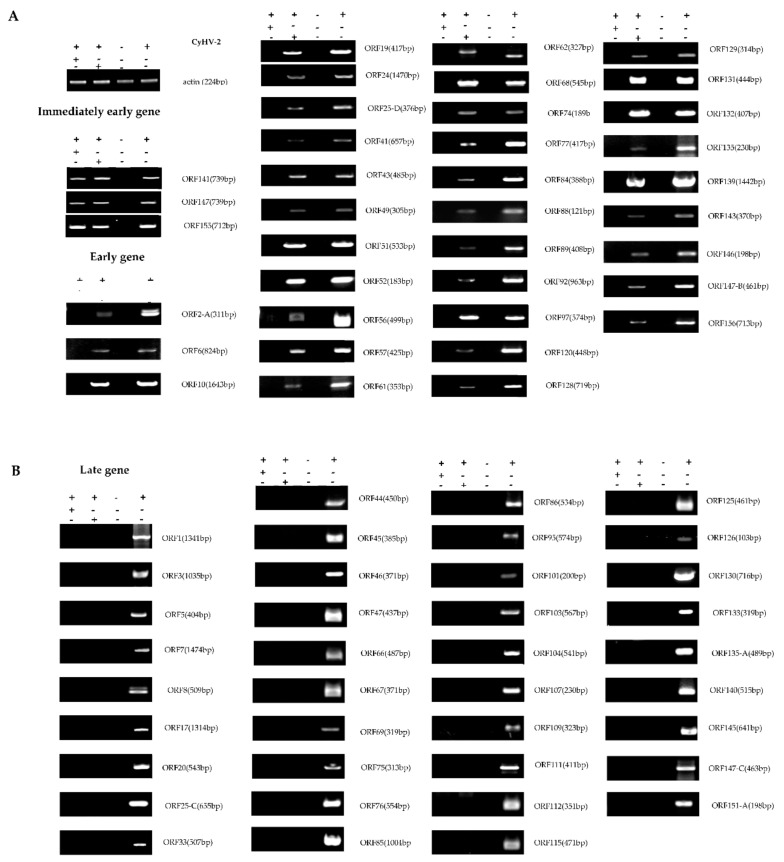
Classification of gene expression distinct phases of CyHV-2 base on inhibitors treatment. According to the transcriptome results, the gene expression in different inhibitor treatment groups were validated by RT-PCR. Specific primers for different ORFs were designed to detect CyHV-2 gene expression and classify the phases of CyHV-2 gene expression. CHX can inhibit the expression of genes other than the early genes of the virus by inhibiting protein synthesis. Early gene expression cannot be suppressed by Ara-C. The gene encoding actin was used as an internal reference. (**A**) Immediate-early and early genes; (**B**) Late genes. CHX: “+” treated with 12 μg/mL CHX, “-” without CHX; Ara-C: “+” treated with 300 μg/mL Ara-C, “−” without Ara-C; CyHV-2: “+” infected with CyHV-2; “−” uninfected.

Subsequently, the 61 candidate CyHV-2 IE genes and other candidate E and L genes were subjected to qRT-PCR analysis. As shown in Figure 4 and Figure 7, among the 61 candidate viral IE genes from RNA-Seq results, five genes (*ORF54, ORF121, ORF141, ORF147*, and *ORF155*) were detected in the presence of both CHX and Ara-C during CyHV-2 infection. Figure 7A shows that 36 ORFs could be detected in the cells treated with Ara-C but without CHX. Additionally, 39 ORFs were not detected in both the CHX treated and Ara-C treated samples (Figure 7B). Ultimately, the five genes listed above were identified as the CyHV-2 IE genes.

Bioinformatic analysis using the SMART (http://smart.emblheidelberg.de), NCBI (https://www.ncbi.nlm.nih.gov/), and Protein Blast http://blast.ncbi.nlm.nih.gov/Blast.cgi) databases showed that three of the proteins encoded by the CyHV-2 IE genes contained important function motifs. The *ORF54* predicted protein contains a zinc finger motif that suggests a DNA binding function. This gene structure is the same as that found in CyHV-3 *ORF54*. Zinc fingers are a common DNA binding domain found in many transcription factors; the results indicated that ORF54 plays a key role in the regulation of other viral genes or host genes. *ORF121* was predicted to express a member of the GAGA binding protein-like family, which have a zinc-binding DNA interaction domain. Additionally, it contains an AAA domain, which is a potential ATP binding and hydrolysis domain. Thus, viral *ORF121* might generate the energy required for translocation. Furthermore, *ORF141* encodes a CyHV-2 large subunit of ribonucleotide reductase annotated to be involved in nucleotide metabolism, DNA, and RNA. The other two viral IE genes (*ORF147* and *ORF155*) had no known functional domains. The biological function analysis of all 150 viral ORFs is shown in Supplementary Table S1.

### 3.3. IE Gene Temporal Expression during CyHV-2 Infection

**Figure 8:**
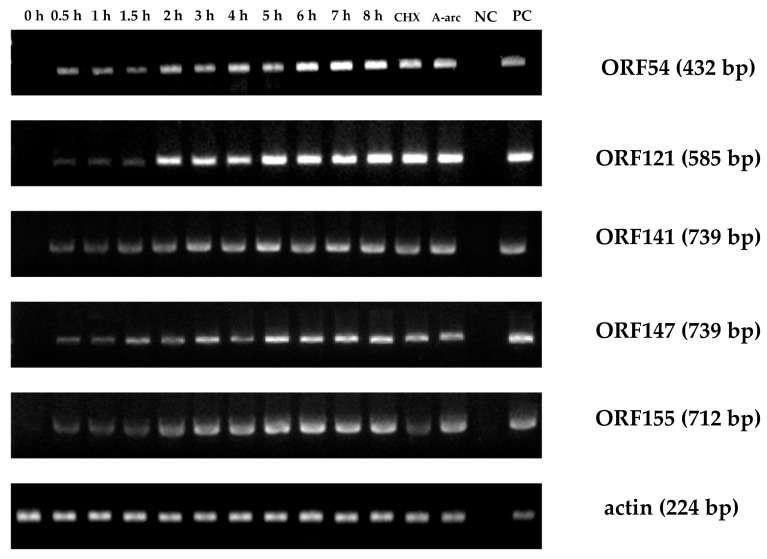
Time course of immediate-early (IE) gene expression during viral infection. At various timepoints, total RNA was extracted from CyHV-2-infected RyuF-2 cells. RT-PCR was used to detect the expression of control genes and all six IE genes. CHX: treated with 12 μg/mL CHX; Ara-C: treated with 300 μg/mL Ara-C; NC: uninfected cells; PC: Positive control comprising CyHV-2 DNA.

**Figure 9:**
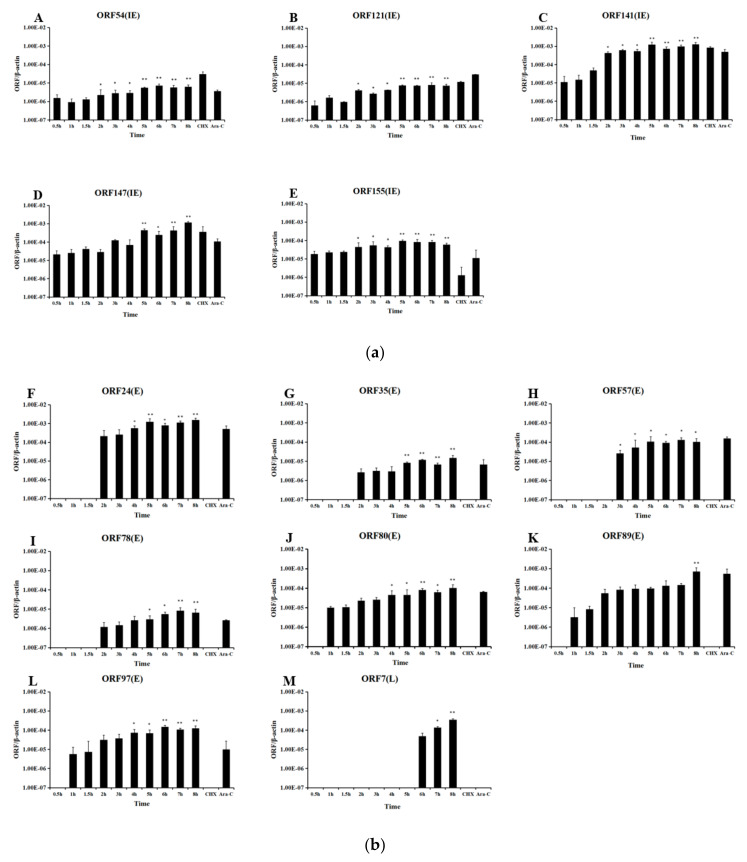
Quantification of CyHV-2 ORF transcripts at indicated time points. Cells were infected with CyHV-2 and treated with no inhibitor, CHX at 300 μg/mL CHX or Ara-C at 12 μg/mL Ara-C before RNA extraction and RT-PCR, followed by cDNA synthesis. The amount of amplified cDNA was normalized to that of the actin mRNA present in the same sample. Genes that were expressed up to 2 h are divided into immediate-early (IE) genes (**A**): *ORF54*, *ORF121*, *ORF141*, *ORF147*, and *ORF155*; genes that begin to be expressed between 2 and 4 h are early (E) genes (**B**): *ORF24*, *ORF35*, *ORF57*, *ORF78*, *ORF80*, *ORF89*, and *ORF97*; and genes that begin to be expressed after 4 h are late (L) genes (**C**): *ORF7.* Error bars represent the standard deviation of the mean of experiments performed in triplicate. o significant difference, * *p* ≤ 0.05 and ** *p* ≤ 0.01.

Cell were sampled at various times post infection, and RNA and cDNA were prepared. Figure 8 shows that all five CyHV-2 IE genes were initially transcribed within 30 min of infection. As determined by qPCR, the expression of all five viral IE genes in CyHV-2-infected cells started at 0.5 h, which is consistent with the qRT-PCR results (Figure 9A). As expected, all the viral IE genes were detected in the CHX and Ara-C treated groups. As controls, qPCR (Figure 9B) showed that the viral E genes (*ORF80*, *ORF89*, and *ORF97*) could be detected at 1 h post-infection, and most of them appeared between 1 and 2 h post-infection. As shown in Figure 9C, viral L genes (*ORF7-1* and *ORF147-C*) appeared at 6 h post-infection. Additionally, as shown in Figure 9B and C, viral E genes could be detected in Ara-C treated samples but not CHX-treated samples, while the viral L genes only appeared in non-treated samples, which was consistent with the previous qRT-PCR results. Consequently, temporal expression analysis clearly distinguished the chronological order of CyHV-2 IE, E, and L gene expression.

## 4. Discussion

Upon infection, viruses with large DNA genomes express their genes according to a tightly regulated cascade, in which numbers of genes are coordinately transcribed. IE gene products are involved in regulating the expression of E and L genes or modulation of host cell function [16]. Therefore, the viral IE genes of DNA viruses play an essential role in viral replication. For example, the capsid protein VP16 of the herpes simplex virus type 1 (HSV-1) could recruit the host transcription machinery to initiate the replication of viral IE genes, such as infected cell protein 0 (ICP0), ICP4, ICP27, and ICP47. ICP0 could induce extensive structural and biological changes to Nuclear domain 10 (ND10). The HSV IE gene product ICP4 acts as a major transcriptional activator that is essential to regulate viral E and L genes. ICP27 promotes viral infection through splicing, processing, and mRNA export [17]. The present study explored CyHV-2 IE genes through high-throughput sequencing combined with the use of inhibitors.

CHX, a protein synthesis inhibitor, has been widely used to identify viral IE genes, such as in CyHV-3, WSSV, and HSV-1 [7,18,19,20,21]. The nucleoside analog Ara-C can be used to differentiate between viral E and L genes [8]. Shibata et al. [22] reported that the RyuF-2 cell line exhibits a higher susceptibility to CyHV-2 infection than other cells; however, EPC, FHM, and BF-2 cells could not be used to propagate the virus, even at a high titer of the CyHV-2. In this study, we employed the CyHV-2-infected RyuF-2 in vitro model to classify the temporal expression patterns of virus genes during infection. Cytotoxicity assays using RyuF-2 cells showed that CHX and Ara-C could be used safely below concentrations of 15 µg/mL and 300 µg/mL. In our previous study, *ORF121* showed the highest expression in almost all tested tissues during viral infection [23]. In previous research on CyHV-3, Ilouze et al. [8] identified *ORF54* as a viral IE gene; *ORF35, ORF72*, and *ORF121* were identified as E genes; and *ORF78* was identified as an L gene. *ORF54, ORF72*, and *ORF78* share around 50% sequence similarity with CyHV-2 and CyHV-3. In addition, CyHV-2 *ORF54* contains a conserved putative zinc-binding domain, as it does in CyHV-3. *ORF72* from CyHV-2 or CyHV-3 encodes the major capsid protein and shows high immunogenicity. Similar to CyHV-3, the capsid protein pORF78 is abundant in mature CyHV-2 particles [24]. In addition, ORF35 is significantly different between CyHV-2 and CyHV-3. Consequently, *ORF54, ORF121, ORF72, ORF78*, and *ORF35* were selected to evaluate the efficiency of the screening model. As shown in Figure 5, the transcription of *ORF54* and *ORF121* were not affected by different concentrations of CHX or Ara-C, classifying them as IE genes. As the CHX concentration in the medium increased, the transcription of *ORF35, ORF72*, and *ORF78* were inhibited (Figure 5). Furthermore, *ORF72*, but not *ORF35* and *ORF78*, could be inhibited by Ara-C at 300 μg/mL. Taken together with the results described above, we concluded that *ORF35* and *ORF78* should be classified as E genes, and *ORF72* should be classified as an L gene. Consequently, CHX at 12 μg/mL and Ara-C at 300 μg/mL could be used to distinguish the CyHV-2 IE, E, and L genes. Table 2 shows that all 150 ORFs of CyHV-2 annotated previously [25] were identified in the control group. The high-throughput sequencing detected 61 ORFs in the CHX treatment group and 127 ORFs in Ara-C treatment group. Finally, temporal expression analysis and qRT-PCR identified 5 IE, 34 E, and 39 L genes (Figure 7 and Figure 8).

In this study, *ORF54, ORF121, ORF141, ORF147*, and *ORF155* were identified as viral IE genes. Several lines of evidence support this conclusion. First, the mRNAs for these five ORFs were detected in the groups with blocked viral protein synthesis (Table 2, Figure 5 and Figure 7), which is a characteristic of viral IE genes. Second, the ORFs were first transcribed earlier than the other viral ORFs (Figure 8). Previous work has shown that *ORF121* is the most abundant protein in moribund fish infected with CyHV-2 [26]. Similarly, *ORF121* was also one of the most highly expressed ORFs during CyHV-3 infection in an acute infection group [27]. As shown in Figure 8B, the transcription of the IE gene *ORF121* started earlier than that of the other viral non-IE genes. This suggested that *ORF121* might exert regulatory effects on viral non-IE genes or host innate immunity genes. Similarly to CyHV-3, *ORF54* and *ORF155* were classified as viral IE genes. As shown in Table 2, *ORF54* contains a putative zinc-binding domain, suggesting it plays a role in regulating other viral or host genes; however, no functional information was available for *ORF155*. *ORF141* encodes a subunit of ribonucleotide reductase that might be essential for DNA synthesis. In addition, Figure 8E-L shows that the transcription of viral E genes started at around 2 h post infection. As shown in Figure 8M,N, viral L genes appeared at 6 h post infection. This suggested that the replication of CyHV-2, like other large DNA viruses, starts with viral IE gene expression, followed by the expression of E and L genes. Large DNA viruses typically carry hundreds of genes, and IE genes encode proteins required for the regulation of viral gene expression.

Among the five CyHV-2 IE genes, two (*ORF54* and *ORF155*) are consistent with the CyHV-3 genes annotated by Ilouze et al. [8]. The other three CyHV-2 IE genes (*ORF121*, *ORF141*, and *ORF147*) identified in this study were different from those of CyHV-3. These three viral genes were considered as E genes in CyHV-3. Moreover, 10 out of the 15 CyHV-3 IE genes were confirmed to have different temporal expression patterns from those of CyHV-2, including *ORF1L/R, ORF3L/R, ORF 6 L/R, ORF7L/R, ORF8L/R, ORF10, ORF24, ORF88*, and *ORF112*. All these ten CyHV-3 IE genes contain different functional domains from CyHV-2, based on the bioinformatic analysis. The other three CyHV-3 IE genes (*ORF9, ORF11*, and *ORF149*) share no sequence homology with those of CyHV-2.

In future studies, the function of viral IE genes should be explored to fully determine the molecular mechanism of CyHV-2 infection. However, the biological function of viral IE genes in CyHV-2 infection is currently unclear. CyHV-2 can lead to acute infection, persistent infection, and latent infection. A recent report by Wei et al. recommended that a persistent infection could re-emerge as an acute infection under certain conditions, such as a dramatic temperature shift. Currently, the complex molecular mechanisms by which CyHV-2 establishes acute infection, persistent infection, or latent infection are incompletely understood. Taken together, the results of the present study provide important insights into the pathogenesis of CyHV-2. Future studies should investigate the functions of CyHV-2 IE genes, such as *ORF54* and *ORF121*, to provide a deeper understanding of CyHV-2’s biology and to achieve control of the disease.

## Figures and Tables

**Table 1:** Oligonucleotide primers and conditions for PCR and Real-time PCR.

PCR	Gene Name	Nucleotide Sequence (5′–3′)	Amplicon Sizen (bp)		Tm (°C)
1	ORF1	F:CTACAGAAGCCTGCCACC	1341		55
		R:TCCGTGGAGTCTGGTCTG			
2	ORF2-A	F:ACCACCACCTCTGCGAAAC	311		55
		R:ATTGTGCCCTGTGCGTTT			
3	ORF3	F:CGTGTATGGAATCCCTCG	1035		55
		R:TGCCTCTGTGGCTTGTAT			
4	ORF5	F:CGCTGAACCTGAAACCCTCC	404		60
		R:GCGTTCCCTGTGGTTCCTG			
5	ORF6	F:TGGCTTTCTGCTGGAGGTG	824		60
		R:CACCAGCACCAACCACTCA			
6	ORF7	F:CGGTTCTCAGCCAGTTCG	355		60
		R:TGCTTGACCCTCCATCCC			
7	ORF8	F:CAGGGACCAGACCGAAGACA	509		60
		R:CGTCCTCGTCTCCAGGGTCT			
8	ORF10	F:AGCAGCAGGTGGCTTCGGATAG	1643		60
		R:GCGGCGTGGATTGTTGGAGT			
9	ORF16	F:CAAGGGTTCAAGAAAGTAA	537		50
		R:TTGGAGCGTCTATGGTAT			
10	ORF17	F:CTCGGCGTAGATGTAAGTG	1314		50
		R:GCCTCTGTAACGGATGAA			
11	ORF19	F:CGAAGGAAACCTGGGAGC	471		60
		R:CGGGCTCGCTTGTTGACT			
12	ORF20	F:GATGCTGTTGCCGACCTT	543		55
		R:TGCGACGTTACCGACCTA			
13	ORF24	F:CGAGGGACTCCACAACCG	470		58
		R:TCAAATCACCCAGACAATACCA			
14	ORF25-C	F:CACCCTACCCTCCGAAAT	655		55
		R:TGAACCGCAATACACCTT			
15	ORF25-D	F:AACCAGACCATCACTCGCATAG	376		60
		R:CCACCACTTGGGCTCCTC			
16	ORF33	F:AGAGTCCGTGAGTAACCTGTGG	507		60
		R:GCATCCGACACCACCTTG			
17	ORF35	F:GTGATGCCGACAGAGGACA	554		60
		R:TGATACTCGGCTCCCTTCT			
18	ORF41	F:TTCTTCTGACGCCAACCA	657		58
		R:CACGGCTTGGGTAGGTTG			
19	ORF43	F:CCAAGCCGTGCGACATAG	485		58
		R:GTTGCCGCCAAACATTAC			
20	ORF44	F:GCGGAGATAATGGAGGTGA	450		55
		R:CAGGAGTCGGGTTGTTGC			
21	ORF45	F:CCACGGTCAAGCAGGGTA	385		55
		R:GCGAGGTCGTCGAAATCA			
22	ORF46	F:ATGGGCGTCGTGGATTAC	371		55
		R:TTCTTGCTGCTGCTGTCG			
23	ORF47	F:TGAATACGCATCCAACTG	437		50
		R:CGTCCATAGAATCCAACC			
24	ORF49	F:GTGGCGGTAGATGCTTTC	305		55
		R:CAGGGTCTGATGGTTGGA			
25	ORF50	F:AAGTCGGCAAGGTGTTTC	341		55
		R:CTTTTGGCGTCTGATGTG			
26	ORF51	F:ACTCGCAGGTGTACTTCAAGGG	533		60
		R:GGCGACGCTGATGAACACG			
27	ORF52	F:CTACTACGCTTCTACTGCTCT	183		50
		R:GTGTCGTTGGGTCCTCTT			
28	ORF54	F:AGGGATGGCCGTCTAGTTTT	432		55
		R:TCGTTGTCGTTGACGAAGAG			
29	ORF56	F:AGTCCATTTACGACGCTA	499		50
		R:TGGTGAAACGATTTAGGA			
30	ORF57	F:CGAGTTTCCGCCCTACAA	425		58
		R:CCCAGTGCTGCCAGTGAT			
31	ORF61	F:CGGCATCCAACACCCTAC	353		55
		R:GAACGAAAGCAGCAGCAA			
32	ORF62	F:CTCCCATTTCCAACCACTC	327		55
		R:CCTCGTAGACCGACTCCTG			
33	ORF66	F:CTCAAGGATTTCAGGACG	487		50
		R:TGATGTAAGGGTTGGTGAC			
34	ORF67	F:CACCCAGACTATTTTACGC	371		50
		R:GCCACTATTGTAGACGATGT			
35	ORF68	F:GGACATACGAAAGGCTACG	545		58
		R:TTGCGGAGGCTGTGACTT			
36	ORF69	F:GCCACCTACCTAAACGAGC	319		55
		R:CGTGTTGAGGAAAGCGAGT			
37	ORF72	F:ACGACCAGAAGGACACCAAG	429		55
		R:GGGGTGGGTAGAGAGGCTAC			
38	ORF74	F:CCGTGATGAACTTGCTGTT	189		55
		R:GGTACGATTCGGAAGGTAGA			
39	ORF75	F:CGTCTACTATCAGCCGTGTC	313		55
		R:GCAATAGGTGGTGAGTTCC			
40	ORF76	F:CAGTTTTCGGTTGGTGTTGAGTTGG	554		55
		R:TTCTGAACGATGATATGAGCGTGAC			
41	ORF77	F:ACGCTGAACATTTCCACTC	417		50
		R:CGTCCTCGTCCTCTTTGA			
42	ORF78	F:AGGATTCCCAAACAAAGCC	462		60
		R:GTCATCGTCGGTGAGTTCC			
43	ORF84	F:GCTCAGGGTCACCAATCA	388		58
		R:ATCCCCGAGTCCGTTCTT			
44	ORF85	F:CACCAGCGGTAGAAGTCG	1004		55
		R:CCACCCATTGTAATAAACG			
45	ORF86	F:ATCATCCCTTTCCCAGACA	534		55
		R:GCAAACCGTCCTCAAAGA			
46	ORF88	F:CTGCTGCCGCTGTTTACC	121		58
		R:TCGCATCCTCGCACTGAC			
47	ORF89	F:ATGGCGGTGGCATTCCTA	408		60
		R:ATTCGGCGACTGACTTGG			
48	ORF92	F:GAGCCACCTTCCTGTTCC	963		55
		R:CGTAGAAGAGGGACGAGTG			
49	ORF95	F:CACTGGGCAGAGGTTGAG	574		55
		R:CAAGTGGTGATCCTGGTCT			
50	ORF97	F:GACGAGCGATGAATACGG	574		55
		R:GTCTCCGTTGATGGGTCT			
51	ORF101	F:CGATGACAAGCCTACCGC	200		58
		R:GAGCCAGCCAGTCGTTGA			
52	ORF103	F:CCGAACCCGAGTTTTATG	567		55
		R:ACGCCACAAACACGACCT			
53	ORF104	F:GTTACTTTCAACCGAGACGC	541		55
		R:GCCGTCGTTACTGCTGAT			
54	ORF107	F:AACAGGACTGGGAGTTTG	230		50
		R:GCGACTGAATATGGGATG			
55	ORF109	F:GGTGCGTCCGACTGGTAGA	323		58
		R:ATGGAGGCGGTGTTCAGC			
56	ORF111	F:AGATTTGGCGAACGAGGAG	411		60
		R:CGTGTTTGTCGGGCAGGT			
57	ORF112	F:CAACGAATTGACGGCTAA	351		55
		R:ATGGACCAGAGTGGGAGT			
58	ORF114	F:CCGTCAACGAGTATTCCA	585		50
		R:CCAGCGTATAGACCCAGA			
59	ORF115	F:TCGGCGAAGAGTCAAAGG	471		55
		R:CCTGCCACAGCGTAAACA			
60	ORF120	F:GGTGATTGTAGCCAACGAG	448		55
		R:TTAGAACGACGGTCTGTGAT			
61	ORF121	F:GCTACTACCACCGTTGTTCCA	585		55
		R:ATCGCTACCATCTTTCTCCTTCT			
62	ORF125	F:GAGAAAGCAGGTCCGAGTT	461		55
		R:ATCCTCTGGTAGCGTGGG			
63	ORF126	F:TACGCTACAACGGACAAG	101		50
		R:ATCGCCCAGATCAAAGAC			
64	ORF128	F:CCACTGCTACCGCTTCTA	719		50
		R:GGCGTACACTCTACCGTCT			
65	ORF129	F:GTTGGGCTGGCATTGTAT	314		55
		R:CGTTAGAGGTCGGTTCTTTG			
66	ORF130	F:CACCAAACCCGCCTCATA	716		55
		R:GTATTATTCTGGGTTTGCTTGG			
67	ORF131	F:GAAGACACGGTCCAAGCG	444		58
		R:CGAGACTCCGATGATTCCTAC			
68	ORF132	F:ACCATCCGTCGTCAACCA	407		55
		R:AAGCGGTATTGTCGTAGCC			
69	ORF133	F:TAGGCTCATCAGGCACAT	319		55
		R:GAAGCAGGACAAGACCAAA			
70	ORF135	F:TCCCAGCCCAACAGACAG	230		55
		R:CCAGAACCCAGGACGAGA			
71	ORF135A	F:CACCCAAGGACCCAGAAC	489		55
		R:CGTCTACGCATCTCAAAGC			
72	ORF139	F:GAGGTGCGGAGAAGTCAG	1442		55
		R:TCTTGTCCTTATCTCCCAGT			
73	ORF140	F:TGATCCGTTCAAAGATAGA	515		50
		R:TGATCCGTTCAAAGATAGA			
74	ORF141	F:ATCCGCTTCAAGTGGCTAA	739		58
		R:GCGTTGAAGATGCGAAGG			
75	ORF143	F:GAGCAGGAGTAGCAGGGTC	370		55
		R:GCTGATGCGTCTGAGAAT			
76	ORF145	F:TGGCAGTGTCTGTCTTGTC	641		50
		R:AGATTATACGCCCTTTGG			
77	ORF146	F:GCGTATGGCGTAGGTAGG	198		55
		R:TGCGAGGATGATGAAGTG			
78	ORF147	F:ATCCGCTTCAAGTGGCTAA	739		58
		R:GCGTTGAAGATGCGAAGG			
79	ORF147B	F:GAGTGGCTGTGGCTGATG	461		55
		R:GTTGCGGTTGTTTGTCTT			
80	ORF147C	F:GAGTGGCTGTGGCTGATG	463		55
		R:ACGTTGCGGTTGTTTGTC			
81	ORF151A	F:ACAGCGAATGGTTGGAAA	198		55
		R:AACGGTTGAAATGGACAGG			
82	ORF155	F:AAAACTCTGCTATGCGAAAT	712		55
		R:TGAGGAATGGCTTGACTG			
83	ORF156	F:ACACGCTACCACCGCCTAC	713		58
		R:CAGCAGCAGCCGTTGAGT			
84	actin	F:CACTGTGCCCATCTACGAG	224		55
		R:CCATCTCCTGCTCGAAGTC			
**RT-PCR**	**Gene Name**	**Nucleotide Sequence (5′–3′)**	**Amplification Efficiency (%)**	**r^2^**	**Amplicon Size (bp)**
1	actin	F:CACTGTGCCCATCTACGAG	97.6	0.9902	224
		R:CCATCTCCTGCTCGAAGTC			
2	ORF7-1	F:TGATGACATCGTCCCCCTCT	101.5	0.9872	149
		R:TATGGCTGTGCCTCAACGAG			
3	ORF24	F:TCGACAAGGTCGAAGTGGTG	98.7	0.9972	84
		R:TGCGAACCACTCCTGTAACC			
4	ORF35	F:GTGATGCCGACAGAGGACA	101.8	0.9880	553
		R:TGATACTCGGCTCCCTTCT			
5	ORF54	F:CAAGATTGCATCGAGCGTGG	109.6	0.9887	281
		R:TGTCATCGTCGTCCGCATAG			
6	ORF57	F:GATTCGGGCATCAGAAACGC	96.6	0.9988	106
		R:GCCCATCACGTAGTAGGCTC			
7	ORF78	F:AGGATTCCCAAACAAAGCC	99.0	0.9903	482
		R:GTCATCGTCGGTGAGTTCC			
8	ORF80	F:TCTGGATCTCGTCGAAAGCG	91.6	0.9932	107
		R:GCCCAGCCTGAGAAAGAACT			
9	ORF89	F:CCTGCCCATAAAGGAACGGT	107.8	0.9981	88
		R:GAGCTCGCGTCCATTATCCA			
10	ORF97	F:CGAGACGCGTACGATAACCA	113	0.9962	147
		R:AACGCTTTCAAAACGGGACG			
11	ORF121	F:GGACATCAAATCGGCAGCTC	101.4	0.9938	193
		R:CTCCTCCATGGTCACATCGG			
12	ORF141	F:CAGTGGCTCCCGTACAGTTT	100.6	0.9969	114
		R:TCGATTGCTTCTTGGGGCTT			
13	ORF147	F:GTCCTGTCAGTGTGGTAGCC	97.1	0.9991	172
		R:GTCCATACAGCTGTGGTGCT			
14	ORF147-C	F:TCAACCTGCTCGTGTCACTC	97	0.9823	229
		R:ACCGTTGCATTACAGTCCGT			
15	ORF155	F:TCAAGCTGTACTCGTGGCTG	93.5	0.9936	73
		R:GAAGTGACACACCACAACGC			
					

**Table 2:** Read quality and mapping results for High-throughput sequencing. Group “1”was infected with CyHV-2. Group “A” was treated with 300 μg/mL A-arc and infected with CyHV-2. Group “C” was CyHV-2 infected, treated with 12 μg/mL CHX inhibition.

Sample	NCBI BioSample	Treatment	Total Reads	Cleaned Reads	Mapped to CyHV-2 Genome
1	SAMN14501569	infectin	98539532	98128002	26583(0.2697%)
A	SAMN14501570	A-arc	100866928	100455032	5887(0.1253%)
C	SAMN14501571	CHX	46974030	46710488	2969(0.0029%)

## Supplementary Materials

The following are available online at https://www.mdpi.com/1999-4915/12/9/994/s1. Supplementary data included for this article can viewed online at https://www.ncbi.nlm.nih.gov/sra/?term=PRJNA621366. Table S1: characteristics of CyHV-2 gene.

## References

[1] Jung S.J., Miyazaki T. (1995). Herpesviral Hematopoietic Necrosis of Goldfish, Carassius-Auratus (L). J. Fish Dis..

[2] Goodwin A.E., Merry G., Sadler J. (2006). Detection of the herpesviral hematopoietic necrosis disease agent (Cyprinid herpesvirus 2) in moribund and healthy goldfish: Validation of a quantitative PCR diagnostic method. Dis. Aquat. Org..

[3] Jeffery K.R., Bateman K.S., Bayley A., Feist S.W., Hulland J., Longshaw C., Stone D., Woolford G., Way K. (2007). Isolation of a cyprinid herpesvirus 2 from goldfish, Carassius auratus (L.), in the UK. J. Fish Dis..

[4] Wu T., Ding Z., Ren M., An L., Xiao Z., Liu P., Gu W., Meng Q., Wang W. (2013). The histo- and ultra-pathological studies on a fatal disease of Prussian carp (Carassius gibelio) in mainland China associated with cyprinid herpesvirus 2 (CyHV-2). Aquaculture.

[5] Stephens F., Raidal S.R., Jones B. (2004). Haematopoietic necrosis in a goldfish (Carassius auratus) associated with an agent morphologically similar to herpesvirus. Aust. Vet. J..

[6] Reed A.N., Izume S., Dolan B.P., LaPatra S., Kent M., Dong J., Jin L. (2014). Identification of B Cells as a Major Site for Cyprinid Herpesvirus 3 Latency. J. Virol..

[7] Yuan Y. (2005). Identification and characterization of herpesviral immediate-early genes. Adv. Struct. Saf. Stud..

[8] Ilouze M., Dishon A., Kotler M. (2012). Coordinated and sequential transcription of the cyprinid herpesvirus-3 annotated genes. Virus Res..

[9] Zeng X.-T., Chen Z.-Y., Deng Y.-S., Gui J., Zhang Q.-Y. (2016). Complete genome sequence and architecture of crucian carp Carassius auratus herpesvirus (CaHV). Arch. Virol..

[10] Ito T., Kurita J., Ozaki A., Sano M., Fukuda H., Ototake M. (2013). Growth of cyprinid herpesvirus 2 (CyHV-2) in cell culture and experimental infection of goldfish Carassius auratus. Dis. Aquat. Org..

[11] Davison A.J., Kurobe T., Gatherer D., Cunningham C., Korf I., Fukuda H., Hedrick R.P., Waltzek T.B. (2012). Comparative Genomics of Carp Herpesviruses. J. Virol..

[12] Fang Q., Seng E.K., Ding Q.Q., Zhang L.L. (2008). Characterization of infectious particles of grass carp reovirus by treatment with proteases. Arch. Virol..

[13] Grabherr M.G., Haas B.J., Yassour M., Levin J.Z., Thompson D.A., Amit I., Adiconis X., Fan L., Raychowdhury R., Zeng Q. (2011). Full-length transcriptome assembly from RNA-Seq data without a reference genome. Nat. Biotechnol..

[14] Langmead B., Salzberg S.L. (2012). Fast gapped-read alignment with Bowtie 2. Nat. Methods.

[15] Kumar S., Stecher G., Tamura K. (2016). MEGA7: Molecular Evolutionary Genetics Analysis Version 7.0 for Bigger Datasets. Mol. Biol. Evol..

[16] Li D.F., Zhang M.C., Yang H.J., Zhu Y.B., Xu X. (2007). Beta-integrin mediates WSSV infection. Virology.

[17] Wang X., Hennig T., Whisnant A.W., Erhard F., Prusty B.K., Friedel C.C., Forouzmand E., Hu W., Erber L., Chen Y. (2020). Herpes simplex virus blocks host transcription termination via the bimodal activities of ICP27. Nat. Commun..

[18] Hagglund R., Roizman B. (2004). Role of ICP0 in the Strategy of Conquest of the Host Cell by Herpes Simplex Virus 1. J. Virol..

[19] Li F., Li M., Ke W., Ji Y., Bian X., Yan X. (2009). Identification of the immediate-early genes of white spot syndrome virus. Virology.

[20] Lin F., Huang H., Xu L., Li F., Yang F. (2011). Identification of three immediate-early genes of white spot syndrome virus. Arch. Virol..

[21] Liu W.-J., Chang Y.-S., Wang C.-H., Kou G.-H., Lo C.-F. (2005). Microarray and RT-PCR screening for white spot syndrome virus immediate-early genes in cycloheximide-treated shrimp. Virology.

[22] Shibata T., Nanjo A., Saito M., Yoshii K., Ito T., Nakanishi T., Sakamoto T., Sano M. (2015). In vitro characteristics of cyprinid herpesvirus 2: Effect of kidney extract supplementation on growth. Dis. Aquat. Org..

[23] Wang H., Xu L., Lu L. (2015). Detection of cyprinid herpesvirus 2 in peripheral blood cells of silver crucian carp, Carassius auratus gibelio(Bloch), suggests its potential in viral diagnosis. J. Fish Dis..

[24] Gao W., Wen H., Wang H., Lu J., Lu L., Jiang Y. (2020). Identification of structure proteins of cyprinid herpesvirus 2. Aquaculture.

[25] Davidovich M., Dishon A., Ilouze M., Kotler M. (2007). Susceptibility of cyprinid cultured cells to cyprinid herpesvirus 3. Arch. Virol..

[26] Xu L., Podok P., Xie J., Lu L. (2014). Comparative analysis of differential gene expression in kidney tissues of moribund and surviving crucian carp (Carassius auratus gibelio) in response to cyprinid herpesvirus 2 infection. Arch. Virol..

[27] Neave M.J., Sunarto A., McColl K.A. (2017). Transcriptomic analysis of common carp anterior kidney during Cyprinid herpesvirus 3 infection: Immunoglobulin repertoire and homologue functional divergence. Sci. Rep..

